# Exploring Resilience in Older African Americans With Advanced Chronic Kidney Disease

**DOI:** 10.1093/geroni/igaf122.1365

**Published:** 2025-12-31

**Authors:** Tyrone Hamler, Seungjong Cho, Tabitha Pederson, Pilar Ingle

**Affiliations:** University of Denver, Denver, Colorado, United States; Texas Tech University, Lubbock, Texas, United States; University of Denver, Denver, Colorado, United States; University of Denver, Denver, Colorado, United States

## Abstract

Chronic kidney disease (CKD) affects about 1 in 7 individuals in the U.S., with prevalence increasing with age. Nearly 50% of those over 75 have CKD. Beyond age, racial disparities impact disease progression, with Black individuals nearly four times more likely to develop kidney failure than Whites. This study examined resilience among older African Americans with advanced CKD. While resilience is recognized as a positive adaptation to adversity, researchers highlight the need to explore contextual factors influencing resilience, as chronic social stressors can negatively affect health. No known studies have examined resilience in older Black adults with CKD. Data were drawn from a larger study on decisional conflict in CKD among older Black adults recruited from an outpatient nephrology clinic in the Midwest. Among participants (N = 124), resilience levels were typical (M = 3.10, SD = .56). At the bivariate level, resilience correlated with more comorbidities (r = .20, p < .001). An OLS regression confirmed this association, while resilience was unrelated to other clinical or demographic variables. These findings suggest that resilience may be a response to managing multiple health conditions rather than a protective factor against comorbidities. Future research should explore mechanisms underlying resilience and their implications for health outcomes. Understanding these relationships can inform targeted interventions that foster resilience while addressing the unique challenges faced by older Black adults with CKD. Addressing both social and clinical determinants of health is critical in addressing disparities and improving well-being in this population.

